# Hematopoietic Cell Transplantation Cures Adenosine Deaminase 2 Deficiency: Report on 30 Patients

**DOI:** 10.1007/s10875-021-01098-0

**Published:** 2021-07-29

**Authors:** Hasan Hashem, Giorgia Bucciol, Seza Ozen, Sule Unal, Ikbal Ok Bozkaya, Nurten Akarsu, Mervi Taskinen, Minna Koskenvuo, Janna Saarela, Dimana Dimitrova, Dennis D. Hickstein, Amy P. Hsu, Steven M. Holland, Robert Krance, Ghadir Sasa, Ashish R. Kumar, Ingo Müller, Monica Abreu de Sousa, Selket Delafontaine, Leen Moens, Florian Babor, Federica Barzaghi, Maria Pia Cicalese, Robbert Bredius, Joris van Montfrans, Valentina Baretta, Simone Cesaro, Polina Stepensky, Neven Benedicte, Despina Moshous, Guillaume Le Guenno, David Boutboul, Jignesh Dalal, Joel P. Brooks, Elif Dokmeci, Jasmeen Dara, Carrie L. Lucas, Sophie Hambleton, Keith Wilson, Stephen Jolles, Yener Koc, Tayfun Güngör, Caroline Schnider, Fabio Candotti, Sandra Steinmann, Ansgar Schulz, Chip Chambers, Michael Hershfield, Amanda Ombrello, Jennifer A. Kanakry, Isabelle Meyts

**Affiliations:** 1 Department of Pediatrics, Division of Pediatric Hematology and Oncology, Bone Marrow Transplant Unit, King Hussein Cancer Center (KHCC), P.O Box 1269, Amman, 11941 Jordan; 2 Department of Pediatrics, ERN RITA Core Center, University Hospitals Leuven, Herestraat 49, 3000 Leuven, Belgium; 3 Department of Microbiology, Immunology and Transplantation, Laboratory for Inborn Errors of Immunity, University Hospitals Leuven, Herestraat 49, 3000 Leuven, Belgium; 4 Department of Pediatric Rheumatology, Hacettepe University, Ankara, Turkey; 5 Hacettepe University Vasculitis Research Center, Ankara, Turkey; 6 Department of Pediatric Hematology, Research Center for Fanconi Anemia and Other Inherited Bone Marrow Failure Syndromes, Hacettepe University, Ankara, Turkey; 7 Division of Pediatric Hematology and Oncology, Bone Marrow Transplant Unit, University of Health Sciences, Ankara City Hospital, Ankara, Turkey; 8 Department of Medical Genetics, Hacettepe University, Sihhiye, 06100 Ankara, Turkey; 9 Division of Pediatric Hematology, Oncology and Stem Cell Transplantation, Helsinki University Hospital, Helsinki, Finland; 10 Pediatric Hematology, Oncology and Stem Cell Transplantation, Children and Adolescents, Helsinki University Hospital, Helsinki, Finland; 11 Institute for Molecular Medicine Finland, HiLIFE, University of Helsinki, Helsinki, Finland; 12 Centre for Molecular Medicine Norway, University of Oslo, Oslo, Norway; 13 Experimental Transplantation and Immunotherapy Branch, National Cancer Institute of the National Institutes of Health, Bethesda, MD USA; 14 Immune Deficiency Cellular Therapy Program, CCR, NCI, MD Bethesda, USA; 15 Laboratory of Clinical Infectious Diseases, National Institute of Allergy and Infectious Diseases, Bethesda, MD USA; 16 Cell and Gene Therapy, Baylor College of Medicine, Houston, TX USA; 17 Cincinnati Children’s Hospital Medical Center, Cincinnati, OH USA; 18 University of Cincinnati College of Medicine, Cincinnati, OH USA; 19 Division of Pediatric Stem Cell Transplantation and Immunology, University Medical Center Hamburg-Eppendorf, Hamburg, Germany; 20 Department of Pediatric Oncology, Hematology and Clinical Immunology, Center for Child and Adolescent Health, Medical Faculty, Heinrich-Heine-University, Düsseldorf, Germany; 21 San Raffaele Telethon Institute for Gene Therapy (TIGET), Pediatric Immunohematology and Bone Marrow Transplantation Unit, IRCCS San Raffaele Scientific Institute Milan, Milan, Italy; 22 Pediatric Immunohematology and Bone Marrow Transplantation Unit, IRCCS San Raffaele Scientific Institute, Milan, Italy; 23 Department of Pediatrics, Willem-Alexander Children’s Hospital, Leiden University Medical Center, Leiden, Netherlands; 24 Department of Pediatric Immunology and Infectious Diseases, Wilhelmina Children’s Hospital, University Medical Centre Utrecht, Utrecht, Netherlands; 25 Pediatric Hematology Oncology, Department of Mother and Child, Azienda Ospedaliera Universitaria Integrata, Verona, Italy; 26 Department of Bone Marrow Transplantation and Cancer Immunotherapy, Hadassah University Medical Center, Jerusalem, Israel; 27 Pediatric Immunology, Hematology and Rheumatology Unit, Hôpital Necker-Enfants Malades, APHP, Paris, France; 28 Department of Internal Medicine, University Hospital Estaing, CHU Clermont-Ferrand, Clermont-Ferrand, France; 29 Clinical Immunology Department, Hospital Saint Louis, Université de Paris, Paris, France; 30 Rainbow Babies and Children’s Hospital, Case Western Reserve University, Cleveland, OH USA; 31 Department of Immunobiology, Yale University School of Medicine, New Haven, CT USA; 32 Department of Pediatrics, University of New Mexico, Albuquerque, NM USA; 33 Department of Pediatrics, Division of Allergy, Immunology, Blood and Marrow Transplantation, University of California San Francisco, San Francisco, CA USA; 34 Newcastle University Translational and Clinical Research Institute and Great North Children’s Hospital, Newcastle Upon Tyne Hospitals NHS Foundation Trust, , Newcastle Upon Tyne, UK; 35 Department of Hematology, University Hospital of Wales, Cardiff, UK; 36 Immunodeficiency Centre for Wales, University Hospital of Wales, Cardiff, UK; 37 Stem Cell Transplant Unit, Medicana International, Istanbul, Turkey; 38 Division of Hematology/Oncology/Immunology, Gene Therapy, and Stem Cell Transplantation, University Children’s Hospital Zurich – Eleonore Foundation & Children’s Research Center (CRC), Steinwiesstrasse 75, CH-8032 Zurich, Switzerland; 39 Pediatric Immuno-Rheumatology of Western Switzerland, Department Women-Mother-Child, Lausanne University Hospital, Lausanne, Switzerland; 40 Division of Immunology and Allergy, Lausanne University Hospital and University of Lausanne, Lausanne, Switzerland; 41 Department of Pediatrics, University Medical Center Ulm, Ulm, Germany; 42 Vanderbilt University Medical Center, Nashville, TN USA; 43 Department of Medicine and Biochemistry, Duke University Medical Center, Durham, NC USA; 44 Metabolic, Cardiovascular, and Inflammatory Disease Genomics Branch, National Human Genome Research Institute (NHGRI), Bethesda, MD USA

**Keywords:** Hematopoietic cell transplantation, Deficiency of adenosine deaminase 2, DADA2, Inborn error of immunity, Bone marrow failure, Immunodeficiency, Autoinflammation

## Abstract

**Purpose:**

Deficiency of adenosine deaminase 2 (DADA2) is an inherited inborn error of immunity, characterized by autoinflammation (recurrent fever), vasculopathy (livedo racemosa, polyarteritis nodosa, lacunar ischemic strokes, and intracranial hemorrhages), immunodeficiency, lymphoproliferation, immune cytopenias, and bone marrow failure (BMF). Tumor necrosis factor (TNF-α) blockade is the treatment of choice for the vasculopathy, but often fails to reverse refractory cytopenia. We aimed to study the outcome of hematopoietic cell transplantation (HCT) in patients with DADA2.

**Methods:**

We conducted a retrospective study on the outcome of HCT in patients with DADA2. The primary outcome was overall survival (OS).

**Results:**

Thirty DADA2 patients from 12 countries received a total of 38 HCTs. The indications for HCT were BMF, immune cytopenia, malignancy, or immunodeficiency. Median age at HCT was 9 years (range: 2–28 years). The conditioning regimens for the final transplants were myeloablative (*n* = 20), reduced intensity (*n* = 8), or non-myeloablative (*n* = 2). Donors were HLA-matched related (*n* = 4), HLA-matched unrelated (*n* = 16), HLA-haploidentical (*n* = 2), or HLA-mismatched unrelated (*n* = 8). After a median follow-up of 2 years (range: 0.5–16 years), 2-year OS was 97%, and 2-year GvHD-free relapse-free survival was 73%. The hematological and immunological phenotypes resolved, and there were no new vascular events. Plasma ADA2 enzyme activity normalized in 16/17 patients tested. Six patients required more than one HCT.

**Conclusion:**

HCT was an effective treatment for DADA2, successfully reversing the refractory cytopenia, as well as the vasculopathy and immunodeficiency.

**Clinical Implications:**

HCT is a definitive cure for DADA2 with > 95% survival.

**Supplementary Information:**

The online version contains supplementary material available at 10.1007/s10875-021-01098-0.

## Introduction

In 2014, biallelic deleterious mutations in the cat eye chromosome region 1 gene (*CECR1,* subsequently renamed *ADA2*), encoding adenosine deaminase 2 (ADA2), were reported as the cause of a monogenic inborn error of immunity disease, deficiency of ADA2 (DADA2) (OMIM # 615,688) [1, 2]. The phenotype comprises recurrent fever and vasculopathy, ranging from livedo racemosa and polyarteritis nodosa (PAN) to intracranial vasculopathy with lacunar strokes and hemorrhages [1–3]. Cytopenias, either autoimmune or due to bone marrow failure (BMF), occur in 50% of patients and present as congenital pure red cell aplasia (PRCA), neutropenia, thrombocytopenia, or pancytopenia [4–6]. Immunodeficiency with hypogammaglobulinemia and recurrent viral and bacterial infections and malignant lymphoproliferation (T-large granular lymphocyte leukemia (T-LGL leukemia) and lymphoma) have also been described [5, 7–9]. DADA2 diagnosis is based on an absence or low levels of plasma ADA2 enzymatic activity and the demonstration of biallelic loss-of-function mutations of *ADA2* [10]. The pathophysiology of DADA2 remains unclear. A picture emerges where ADA2 deficiency results in skewing of macrophage differentiation towards inflammatory M1 macrophages [11], leading to endothelial instability, as shown in a zebrafish model and in endothelial cell coculture systems [1]. Recent findings have revealed an even more complex interplay between endothelial cells and monocytes and macrophages with marked ADA2 secretion by endothelial cell lines [12].

Treatment of DADA2 is challenging and case mortality is estimated to be around 8%, mostly in childhood and related to vasculopathy-associated complications and infections [13–15]. None of the classical immunosuppressive drugs are an option for the long-term treatment of DADA2, because their efficacy is temporary, especially for the DADA2 related cytopenia, or because of the toxicity associated with long-term use. Anti-TNF agents, etanercept in particular, are the mainstay of treatment for the inflammatory and vasculopathy phenotypes [16]. However, anti-TNF agents do not cure the hematological phenotype and in a proportion of patients, vasculopathy persists despite anti-TNF treatment [17, 18]. Finally, the cost of life-long TNF-inhibition is a limitation for some patients. Hematopoietic cell transplantation (HCT) has been reported to result in a rapid and sustained resolution of the systemic inflammation and hematological phenotype, with all patients from a cohort of 14 patients surviving after HCT [5, 19–21]. We report here the results of a multinational study of a cohort of 30 patients with DADA2 undergoing HCT, including the previously reported cases.

## Methods

### Overview of the Study

We conducted an investigator-driven retrospective international non-interventional multicenter study on HCT for DADA2. Invitations to participate were sent to the physicians allied to the DADA2 Foundation, the European Group for Blood and Marrow Transplantation (EBMT), and the European Society for Immunodeficiencies (ESID). We also invited all authors of published single case reports on HCT in DADA2 to participate in the study. Data collection began after the Second Inaugural International Conference on DADA2 hosted by the DADA2 Foundation on November 9, 2018. The study was approved by the Ethics Committee of Leuven University Hospitals (study number S63982). The study was performed in accordance with the principles of the Declaration of Helsinki. The authors assume responsibility for the accuracy and completeness of the data and analyses and for fidelity to the study protocol.

### Patients

The criteria for patient inclusion in the study were as follows: (1) genetic diagnosis of DADA2 and/or clinical findings consistent with DADA2 and plasma ADA2 activity level in the deficient range and (2) HCT performed with a follow-up time for survivors of at least 3 months after HCT. All participating physicians completed a questionnaire. All patients or their guardians gave written informed consent for data collection. Patients for whom incomplete data were obtained (indication for HCT, age at HCT, total nucleated cell dose or CD34 + stem cell dose, stem cell donor, conditioning regimen, graft-versus-host disease (GvHD) prophylaxis, time to engraftment, graft failure, conditioning for subsequent HCTs, chimerism) were excluded from the study.

### HCT Data

Neutrophil engraftment was defined as the first of three consecutive days with a neutrophil count ≥ 0.5 × 10^9^/L and platelet engraftment as the first of seven consecutive days with a platelet count ≥ 20 × 10^9^/L without platelet transfusion in the prior 7 days. Full donor chimerism was defined as ≥ 95% donor cells in myeloid or whole-blood fractions. The type of test was at the discretion of the transplant center. Primary and secondary graft failures (GF) were defined according to EBMT guidelines. Second (or third) HCT was defined as the infusion of hematopoietic progenitor cell containing product, according to CIBMTR, regardless of conditioning regimen. The diagnosis and grading of acute and chronic GvHD were based on international standard criteria [22]. Transplant regimen, GvHD prophylaxis, antimicrobial prophylaxis, and pre-emptive treatment were chosen according to center preferences. Preparative regimens were classified as reduced intensity conditioning (RIC) if the dose of alkylating agents or TBI is reduced by at least 30% from a myeloablative conditioning (MAC) approach. A total dose of treosulfan > 30 g/m^2^ was considered MAC whether or not combined with another alkylator, whereas a total busulfan dose < 8 mg/kg and fludarabine-melphalan regimens were considered RIC.

Kaplan–Meier curves were plotted for overall survival (OS) and GvHD-free relapse-free survival (GRFS), and *p* values were obtained for Mantel-Cox log-rank tests performed with Graph-Pad Prism Software version 9. Values of *p* < 0.05 were considered statistically significant. GvHD relapse-free survival was calculated as the time from first HCT until the first occurrence of any of the following events: grades 3–4 aGvHD or moderate/severe cGvHD GF, disease relapse (poor graft function/graft failure with DADA2 disease relapse requiring repeat transplant), or death. HCTs inadvertently using affected donors were excluded. The cumulative incidence of GvHD and GF were also calculated using competing risk analysis, using R, for all HCT procedures, but excluding HCTs from affected donors.

## Results

### Patient Characteristics and Diagnosis of DADA2

We included 30 DADA2 patients undergoing HCT between 2000 and 2020 in this study. Four other patients were excluded due to incomplete data sets. Patients underwent HCT at 21 different centers from 12 countries in Europe and North America. Twenty of the patients have been reported before [4, 5, 7, 9, 19–21, 23–27]. Median age at disease onset was 2.25 years (range: birth to 16 years). Median age at genetic diagnosis was 12 years (range: 2–28 years) (Table 1). DADA2 diagnosis was confirmed at the molecular level in all patients, by demonstrating the presence of biallelic pathogenic *ADA2* variants. Plasma ADA2 activity was assessed before HCT in 18 patients and was low in all cases. Twenty-six patients harbored known pathogenic *ADA2* mutations. The R169Q variant was the most common mutation, found in 15 patients. Four patients harbored novel mutations, all with combined annotation-dependent depletion (CADD) scores above the mutation significance cutoff (MSC) for this gene; all these variants were private or had a MAF < 10^–6^ [28], strongly suggestive of a deleterious effect. Three of these patients were tested for ADA2 enzyme activity, which was found to be low or absent (Table 2). Patient and HCT characteristics are summarized in Table S1 in the Online Supplement.

**Table 1:** Demographic and clinical features of the 30 DADA2 patients before HCT

Patient ID	Sex/Ethnicity	Age at disease onset (y)	Age at genetic diagnosis (y)	DADA2 clinical manifestations	CD4, CD8, CD19, CD56	IgG	IgA	IgM	Previous treatment	Reference
P1^*^	M, Caucasian	0.5	5	RCA, pancytopenia, splenomegaly, recurrent infections, LAP	**653, 93, 11, 0.5**	997	**2.4**	**7**	Prednisone, sirolimus, tacrolimus, IVIG	[5, 19, 21]
P2	F, Tunisian	7	28	PRCA, stroke, EBV viremia, livedo, splenomegaly, aphthous ulcers	**370,84, 60, 48**	1700	60	310	Prednisone, everolimus, hydroxychloroquine, IVIG	[9]
P3	F, Turkish	1	3	PRCA	1460, 690, NA, 255	1200	77	77	Prednisone	[24]
P4	M, Turkish	11	22	PRCA, HSM, MDS-RCMD, recurrent infections, FTT	NA	**682**	**50**	61	Prednisone	[24]
P5	M, Caucasian	0.3	9	PRCA, splenomegaly, IBD, recurrent fevers, aphthous ulcers	**607**, 781, 685, **90**	**290**	119	**9**	Prednisone, anakinra	[21]
P6	M, Caucasian	0	15	Neutropenia, HSM, LAP, livedo, strabismus, PAN, neuropathy	1239, 714, 116, 339	1021	77	149	Prednisone, etanercept, adalimumab, CsA, GCSF	
P7	M, Caucasian	2	22	Anemia, lymphopenia, HSM, livedo, ICH, optic nerve atrophy, PAN	**50, 50, 1, 10**	**465**	**47**	**17**	Prednisone, azathioprine, infliximab, FFP, IVIG	[21]
P8	M, Caucasian	0	2	PRCA, LAP, HSM, recurrent infections, liver fibrosis	1763, 1037, **415, 104**	**426**	** < 8**	** < 6**	Prednisone, IVIG	[21]
P9	F, Caucasian	2.5	4	RCA, pancytopenia, livedo, epilepsy, T-LGL, HSM, aphthous ulcer	**386, 257**, NA, NA	1000	NA	74	Prednisone	[7, 21]
P10	F, Caucasian	7	11	RCA, pancytopenia, AIHA, splenomegaly, ICH, livedo, arthritis	**528, 211, 26, 30**	816	< 26	25	Prednisone, MTX, infliximab, IVIG	[21]
P11	F, Caucasian	1	26	Pancytopenia, HSM, recurrent infections, PAN, bronchiectasis	**183, 108, 0, 2**	700	**42**	**33**	Prednisone, azathioprine, daratumumab, etanercept, CsA, rituximab, IVIG, GCSF	
P12^*^	M, Caucasian	0.4	4	Anemia, neutropenia, HSM, LAP, IBD, SAH, TIA, recurrent infections	**599, 278, 228, 147**	**436**	**17**	54	Prednisone, azathioprine, sirolimus, etanercept, IVIG	[21]
P13	M, Caucasian	4	4	RCA, pancytopenia, FTT, fevers, arthralgia	1926, 2005, **1, 74**	1000	**16**	**10**	Prednisone, etanercept, eltrombopag, infliximab, rituximab, IVIG	
P14	F, Caucasian	12	13	Neutropenia, lymphopenia, recurrent infections, aphthous ulcers	**190, 172, 26, 35**	730	81	**27**	Etanercept	
P15	M, Caucasian	3	4	PRCA, HSM, alopecia, recurrent fevers, strabismus, aphthous ulcers	**814, 459, 104, 60**	605	40	** < 6**	Prednisone, MMF, CsA, sirolimus, IVIG, GCSF	[4, 21]
P16	M, Caucasian	0.1	13	SAA, HSM, livedo, IDDM, GHD, hypothyroidism	1610, 940, 1250, 1040	**360**	60	30	None	[20, 21]
P17	F, Caucasian	0.3	6	PRCA, splenomegaly, recurrent infections, livedo, arthritis, T-LGL	NA	760	116	52	Prednisone, etanercept	
P18	M, Caucasian	0.2	6	RCA, neutropenia, HSM, LAP, portal HTN, hepatoportal sclerosis/fibrosis, recurrent infections	**154, 280**, 225, 224	883	88	60	Prednisone, etanercept, GCSF	
P19	M, Hispanic	14	15	Neutropenia, HSM, NRH	**185, 310, 38, 26**	1247	**20**	**6**	Adalimumab, IVIG, GCSF	
P20	F, Caucasian	16	25	Neutropenia, HSM, recurrent infections, lymphoproliferation	1670, 1721, **106**, 101	NA	NA	NA	Prednisone, ATG, GCSF	[21]
P21	F, Black	12	12	Anemia, neutropenia, LAP, HSM, recurrent infections, EBV, bronchiectasis, DLBCL	**170, 176**, **127, 83**	**580**	49	**31**	Prednisone, rituximab, DLBCL-type chemotherapy	[25]
P22	F, Hispanic	7	21	Neutropenia, recurrent infections	**361, 262, 0, 32**	NA	NA	NA	Prednisone, IVIG, GCSF	[21, 27]
P23	F, Caucasian	3	5	ALPS-like, recurrent infections, neutropenia, splenomegaly, livedo	976, 738, 596, 263	2950	348	**25**	Prednisone, sirolimus, MMF, CsA, GCSF	[23]
P24	F, Algerian	6	NA	Neutropenia, AIHA, HSM, recurrent infections, livedo	1208, 1779, **34**, 134	**225**	**2**	**37**	Prednisone, etanercept, sirolimus, rituximab, IVIG	
P25	F, Turkish	0.3	2	PRCA, HSM, recurrent infections, livedo	**960**, 980, 756, **142**	1000	109	36	Prednisone	[24]
P26	F, Caucasian	2	22	Neutropenia, stroke, T-LGL, recurrent infections, splenomegaly, livedo	**590**, 1450, **121, 6**	**487**	**55**	394	Prednisone, etanercept, hydroxychloroquine	
P27	F, Caucasian	1	9	Pancytopenia, stroke, ICH, livedo, arthritis, AML, HSM, HTN, CMP	**273, 222, 23, 29**	**354**	**34**	**9**	Prednisone, etanercept, anakinra, azathioprine, IVIG	[21, 26]
P28	F, Caucasian	14	15	RCA, neutropenia	**441, 276, 43, 35**	**499**	**11**	**15**	Prednisone, IVIG	[21]
P29*	M, Hispanic	0	18	PRCA, aphthous ulcers, moderate liver siderosis, hepatitis	NA	NA	NA	NA	Prednisone	[21]
P30*	F, Hispanic	13	14	RCA, neutropenia, liver fibrosis, recurrent warts, livedo	**588**, 416, 176, **25**	**625**	86	**10**	Adalimumab, IVIG	

^*^Siblings (1 + 12 and 29 + 30); bold font indicates low values for age. Patients are arranged per donor then age. *AIHA* autoimmune hemolytic anemia, *ATG* antithymocyte globulin, *CMP* cardiomyopathy, *CsA* cyclosporine A, *DLBCL* diffuse large B cell lymphoma, *F* female, *FFP* fresh frozen plasma, *GCSF* granulocyte colony stimulating factor, *GHD* growth hormone deficiency, *HSM* hepatosplenomegaly, *HTN* hypertension, *ICH* intracranial hemorrhage, *IBD* inflammatory bowel disease, *IDDM* insulin-dependent diabetes mellitus, *LAP* lymphadenopathy, *LGL* large granular lymphocyte leukemia, *M* male, *MDS-RCMD* myelodysplastic syndrome-refractory cytopenia with multilineage dysplasia, *MMF* mycophenolate mofetil, *NRH* liver nodular regenerative hyperplasia, *PAN* polyarteritis nodosa, *PRCA* pure red cell aplasia, *RCA* red cell aplasia, *SAH* subarachnoid hemorrhage, *TIA* transient ischemic attack, *URI* upper respiratory infection, *y* year. Lymphocyte subsets (n x 10^6^/L). IgG, IgA, IgM (mg/dL)

**Table 2:** Genetics and ADA2 enzymatic activity for the 30 DADA2 patients

Patient ID	*ADA2* allele 1	*ADA2* allele 2	ADA2 activity pre-HCT	ADA2 activity post-HCT
P1^*^	c.506G > A (p.R169Q)	c.506G > A (p.R169Q)	NA	22.07^a^
P2	c.(753 + 168_754-229)del	c.(1081 + 139_1082-92)del	2^b^	490^b^ at 1y
P3	c.680-681del (p.Y227fs*27)	c.680-681del (p.Y227fs*27)	NA	NA
P4	c.1445 A > G (p.Y482C)	c.1445 A > G (p.Y482C)	NA	44.38^a^
P5	c.144del (p.R49fs)	c.47 + 2 T > C (splice site)	0.2^a^	11.7^a^
P6	c.506G > A (p.R169Q)	c.139G > T (p.G47W)	0.37^a^	1.67 of normal
P7	c.506C > T (p.R169Q)	c.2 T > C (p.M1T)	NA	NA
P8	c.144delG (p.R49fs)	c.506G > A (p.R169Q)	NA	NA
P9	c.506G > A (p.R169Q)	c.506G > A (p.R169Q)	0.0^a^	NA
P10	c.660C > A (p.Y220X)	c.660C > A (p.Y220X)	2.5^b^	403.6^b^ at 2y
P11	**c.3936delG (p.R131Sfs)**	**c.3936delG (p.R131Sfs)**	1.2^b^	NA
P12^*^	c.506G > A (p.R169Q)	c.506G > A (p.R169Q)	0.11^a^	76.5^b^
P13	c.506G > A (p.R169Q)	c.506G > A (p.R169Q)	0^b^	77.8^b^ at 2.5 m
P14	c.506G > A (p.R169Q)	c.506G > A (p.R169Q)	0.09^a^	NA
P15	c.1110C > A (p.N370K)	c.1072G > A (p.G358R)	0.6^a^	19.7^a^ at 1y
P16	c.506G > A (p.R169Q)	c.506G > A (p.R169Q)	NA	8.3^a^ at 10y
P17	c.506G > A (p.R169Q)	c.932 T > G (p.L311R)	0.3^a^	NA
P18	c.336C > G (p.H112Q)	del exon 7	0.4^a^	0.4 of normal
P19	c.506G > A (p.R169Q)	c.336C > G (p.H112Q)	NA	NA
P20	c.140G > T (p.G47V)	c.336C > G (p.H112Q)	NA	NA
P21	c.934C > T (p.R312X)	**c.709delC (p.Glu237fs)**	0.2^a^	35.6^a^ at 2 m
P22	c.794C > G (p.S265X)	c.794C > G (p.S265X)	0.0^a^	10.8^a^ at 1y
P23	c.1367A > G (p.Y456C)	c.1196. G > A (p.W399X)	NA	21.4^a^
P24	c.140G > T (p.G47V)	c.140G > T (p.G47V)	NA	NA
P25	c. 1072 G > A (p.G358R)	c. 1072 G > A (p.G358R)	0.52^a^	NA
P26	**p.Lys188Pro**	**g.17188016_17188596del**	0^a^	6^a^
P27	c.506G > A (p.R169Q)	c.506G > A (p.R169Q)	0.8^a^	7.0^a^ at 1y
P28	**c.144dupG (p.R49fs)**	c.506G > A (p.R169Q)	NA	NA
P29*	c.506G > A (p.R169Q)	c.1072G > A (p.G358R)	NA	22.3^a^
P30*	c.506G > A (p.R169Q)	c.1072G > A (p.G358R)	0.3^a^	NA

^*^Siblings (1 + 12 and 29 + 30)

^a^Plasma ADA2 (mU per mL): healthy controls (*n* = 27 + pooled normal plasma), 13.0 ± 5.1 (4.7–27.2). DADA2 patients (*n* = 55), 0.4 ± 0.5 (0–2.5)

^b^Dried plasma spots ADA2 (mU/g protein): healthy controls (*n* = 106), 130.0 ± 53.2 (24.9–285). DADA2 patients (*n* = 78), 4.7 ± 4.8 (0–23.3)

### Hematological Phenotype Pre-HCT

PRCA was documented in 8/30 patients, isolated neutropenia in 6/30, combined RCA and neutropenia in 5/30, severe aplastic anemia in 1/30, severe lymphopenia in 1/30, anemia and neutropenia in 2/30, autoimmune hemolytic anemia (AIHA) in 2/30, and pancytopenia in 5/30 patients, at presentation. Six patients had a hematological malignancy or myelodysplasia and received HCT as part of the therapeutic approach (P4, P9, P17, P21, P26, and P27). Twenty-nine patients had received at least one immunosuppressive treatment prior to HCT including 13 patients who received at least 3 lines of immunosuppressive medications. Fourteen patients had received anti-TNF agents before HCT, without effect on cytopenias. P6 received single agent etanercept, which failed to reverse neutropenia; a combination of adalimumab, cyclosporine, and low-dose prednisone resulted in normal neutrophil counts for 6 months prior to HCT. Two patients received pre-HCT Interleukin-1 receptor antagonist (anakinra), without amelioration of cytopenias or immune dysregulation. Seven patients received granulocyte colony stimulating factor (G-CSF) for neutropenia, with no response.

### Immunological and Vasculitis Phenotype Pre-HCT

IgG levels were low in 12/27 tested patients, IgA levels were low in 13/26, and IgM levels were low in 15/27 tested patients. Recurrent infections were reported in 17 of the 30 patients, mostly viral infections (in 14/17). Herpesvirus infections predominated, with three patients suffering from recurrent herpes zoster, one having protracted CMV infection, one with severe chicken pox, one with recurrent cutaneous HSV-1, two with HHV6 viremia, and four with EBV viremia (2 transient, 1 chronic, and 1 in the context of lymphoproliferative disease). Warts (*n* = 4), and mollusca contagiosum (*n* = 4) were also reported. Immunoglobulin substitution treatment was administered to 15 of the 30 patients before HCT, and splenomegaly was reported in 23/30 patients. Fifteen of 30 patients had reported vasculitis prior to HCT (Table 1): 9 had livedo racemosa, 3 had polyarteritis nodosa (PAN) (P6 with livedo, P7 with ICH and livedo, P11 isolated), three patients had ischemic stroke (P2, 26, 27), and four had intracranial hemorrhage (P7,10, 27, P12).

### Transplant Characteristics

The indications for HCT were cytopenia with or without immunodeficiency and/or lymphoproliferation or malignancy (Table 3). The median age at HCT was 9 years (range: 2–28). Six of the 30 patients had received HCT before the description of DADA2 in 2014. Two patients (P16, P29) were inadvertently transplanted using affected siblings as donors and received salvage second HCT from unrelated donors. A total of 38 HCTs were performed for 30 patients. Twenty patients received MAC (P12 with two subsequent HCTs), eight received RIC, and two received NMA conditioning for the final curative transplant (Tables 3 and S1). The most commonly used regimen (in 11 patients) was treosulfan/fludarabine ± thiotepa with antithymocyte globulin (ATG) or alemtuzumab. Serotherapy was used in 25/30 patients: ATG in 10 (rabbit ATG in all except horse ATG in P6 and P18) and alemtuzumab in 15 patients. The source of the stem cells for the final transplant was peripheral blood (PB) for 10 and bone marrow (BM) for 20 patients. Donors were HLA-matched related (*n* = 4), HLA-haploidentical sibling (*n* = 2), 10/10 HLA-matched unrelated (*n* = 16), and 9/10 HLA-mismatched unrelated (*n* = 8) for the final transplant (Tables 3 and S1). Two of the six related donors carried heterozygous *ADA2* mutations (P3, P22), three were healthy (P1, P2, P21), and one donor was of unknown status (P4).

**Table 3:** HCT data and post-HCT complications for the 30 DADA2 patients

Patient ID	Year of HCT	Age at HCT (y)/sex	Indication for HCT	HLA match/graft source	Conditioning	GvHD prophylaxis	CD34^+^ dose (× 10^6^/kg)	aGvHD/grade	cGvHD	Other comp	Last donor chimerism	Last follow-up (m)
P1^*^	2009	3/M	RCA, neutropenia	MSD BM	Bu/Cy (MAC)	MMF/CsA	7.5	Gut 3	No	ITP, SOS on D + 60	3 y > 95%	114
P2	2018	28/F	PRCA	MSD BM	Bu/Flu (MAC)	CsA	4.3	No	No	None	2 y 91% (myeloid 100%)	26
P3	2016	3/F	PRCA	MRD BM (ADA2 carrier)	Bu/Flu/TT (MAC)	MTX/CsA	5.4	No	No	None	2 y 100%	48
P4	2016	23/M	PRCA	MRD PB (unknown ADA2 status)	Bu/Flu (MAC)	MMF/PTCy	10.5	Skin 1	Skin + mouth, mild	None	6 m 100%	41
P5	2015	7/M	PRCA	10/10 MUD BM	Bu/Cy/ATG (MAC)	MTX/CsA	TNC = 7.5	No	No	None	1 y 100%	60
P6	2019	17/M	Neutropenia	10/10 MUD PB	Bu/Cy/pentostatin/hATG (RIC)	MMF/Tacro/PTCy	7.7	Skin 1	No	None	1 y 100%	20
P7	2016	23/M	Severe lymphopenia	10/10 MUD BM	Bu/Flu/Alem (MAC)	MTX/CsA	6.4	No	No	None	6 m > 95%	50
P8	2016	2/M	PRCA, recurrent CMV	10/10 MUD BM	Flu/Treo/TT/ATG (MAC)	MTX/CsA	8.3	Skin 2	No	None	6 m 100%	50
P9	2016	5/F	RCA, neutropenia	10/10 MUD BM	Flu/Treo/TT/Alem (MAC)	MMF/CsA	9	Skin 1	No	None	1 y 98%	48
P10	2016	11/F	Pancytopenia, autoimmunity	10/10 MUD PB	Flu/Treo/TT/ATG (MAC)	MTX/CsA	3.2	Skin 1	No	Steatosis hepatis	1 y 100%	50
P11	2019	28/F	Pancytopenia	10/10 MUD BM	Flu/Treo/TT/Alem (MAC)	MMF/CsA	4.8	No	No	None	6 m 100%	12
P12^*^	2016	5/M	Recurrent TIA, immunodeficiency	10/10 MUD PB (2 boosts for declining MC)**	Flu/Treo/Alem (MAC)	MMF/CsA	8.1	Skin 1	No	None	3 y > 95%	54
P13	2020	6/M	PRCA	10/10 MUD BM	Flu/Treo/Alem (MAC)	MMF/Tacro	5.7	No	No	None	6 m 100%	6
P14	2018	14/F	Neutropenia, severe lymphopenia	10/10 MUD PB	Flu/Treo/Alem (MAC)	MMF/CsA	15	Skin 1	No	Mild bronchiectasis	2 y 100%	22
P15	2016	4/M	PRCA, neutropenia	10/10 MUD BM	Flu/Mel/Alem (RIC)	MTX/Tacro	3.2	No	No	ITP D + 42	1 y 98%	52
P16	2003	4/M	Refractory SAA	10/10 MUD BM (1st HCT from affected MSD)	Flu/TBI/Alem (RIC)	MTX/CsA	1.4	No	No	None	3 y 100%	200
P17	2019	6/F	Immune dysregulation	10/10 MUD BM	Flu/Treo/TT/Alem (MAC)	CsA	10.8	Skin 1	Skin, moderate	None	3 m 96%	11
P18.1	2018	7/M	RCA, neutropenia	10/10 MUD PB	Bu/Cy/pentostatin/hATG (RIC)	MMF/Tacro/PTCy	9	No	No	Secondary GF		
P18.2	2019			10/10 MUD PB	Flu/Cy/Alem (NMA)	CsA/PTCy	3.7	No	No	Unstable graft		
P18.3	2019			10/10 MUD PB CD34 selected + 2 week post-HCT DLI	Flu/rATG (NMA)	None	7.4	Skin, liver, gut 2	No	NRH, siderosis	8 m 100%	24
P19.1	2017	19/M	Neutropenia	10/10 MUD BM	Bu/Cy/pentostatin (RIC)	MMF/Siro/PTCy	4.5	No	No	SOS on D + 21, secondary GF		
P19.2	2018			10/10 MUD PB	Flu/Alem (NMA)	CsA	8.5	No	No	Stable NRH	1 y 100%	36
P20	2014	23/F	Neutropenia	10/10 MUD BM	Flu/Mel/Alem (RIC)	Prednisone/CsA	TNC = 2.1	No	No	None	2 y 98%	76
P21	2018	13/F	Diffuse large B-cell lymphoma	Haplo brother PB	Flu/Mel/TT/ATG/rituximab (RIC)	alpha–beta TCD	7	No	No	None	1 y 100%	30
P22	2013	20/F	Neutropenia	Haplo sister BM (ADA2 carrier)	Flu/Bu/Cy/TBI200 (MAC)	Tacro/MMF/PTCy	0.5	No	No	None	3 y 100%	78
P23	2015	5/F	Neutropenia	9/10 MMUD BM	Bu/Flu/TT/ATG/ rituximab (MAC)	MTX/CsA	4.2	Skin/gut 2	Gut, mild	None	1 y 100%	45
P24	2018	9/F	Neutropenia, AIHA	9/10 MMUD BM	Bu/Flu/Alem/Rituximab (MAC)	MMF/CsA/PTCy	5.6	No	No	SOS on D + 12, ARDS D + 54	1 m 100%	2 (dead)
P25	2020	4/F	PRCA	9/10 MMUD PB	Flu/Treo/TT/ATG (MAC)	MTX/CsA	4.5	Skin 2	No	None	1 m 100% to 11 m 55%	12
P26	2018	24/F	Neutropenia	9/10 MMUD BM	Flu/Treo/TT/ATG/ rituximab (MAC)	MTX/CsA	3	No	No	None	2 y 100%	51
P27	2012	8/F	Pancytopenia	9/10 MMUD BM	Flu/Treo/Alem (MAC)	MTX/CsA	8.6	No	No	None	5 y 100%	98
P28.1	2016	16/F	RCA, neutropenia	9/10 MMUD BM	Flu/Treo/TT/ATG (MAC)	MTX/CsA	1.4	No	No	Primary GF		
P28.2				9/10 MMUD PB (different donor)	Flu/TT/ATG (RIC) (alpha–beta TCD)	MTX/CsA	4.9	No	No	None	1 m 100%	42
P29*	2007	9/M	PRCA	9/10 MMUD PB (1^st^ HCT from affected MSD)	Flu/TBI450/Alem (RIC)	MTX/CsA	7.9	Skin 2	Skin + liver moderate	AIHA D + 70 bridging liver fibrosis	3 y 95%	155
P30*	2017	16/F	RCA, neutropenia	9/10 MMUD BM	Flu/Mel/TT/Alem (RIC)	prednisone/CsA	TNC = 2.5	No	Skin mild	Hepatitis	1 y 100%	36

^*^Siblings (1 + 12 and 29 + 30); **unconditioned boosts 1 month apart; *AIHA* autoimmune hemolytic anemia, *Alem* alemtuzumab, *ARDS* acute respiratory distress syndrome, *hATG* horse antithymocyte globulin, *BM* bone marrow, *Bu* busulfan, *comp* complications, *CsA* cyclosporine A, *DLI* donor lymphocyte infusion, *Flu* fludarabine, *GF* graft failure, *GvHD* graft versus host disease, *HCT* hematopoietic cell transplant, *m* month, *MC* mixed chimerism, *Mel* melphalan, *MTX* methotrexate, *MMF* mycophenolate mofetil, *MSD* HLA-matched sibling donor, *MUD* HLA-matched unrelated donor, *MMUD* HLA-mismatched unrelated donor, *PB* peripheral blood, *NRH* nodular regenerative hyperplasia, *PRCA* pure red cell aplasia, *RCA* red cell aplasia, *Siro* sirolimus, *SOS* sinusoidal obstruction syndrome, *TBI* total body irradiation, *TNC* total nucleated cell dose, *Treo* treosulfan, *TT* thiotepa, *PTCy* post-transplant cyclophosphamide, *y* year

### Engraftment, Graft Failure, Transplant-Related Morbidity, Survival

**Fig. 1:**
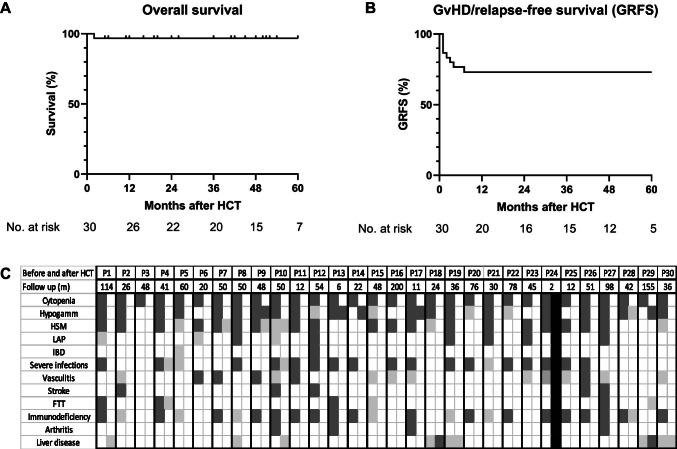
Kaplan–Meier curves representing **A** overall survival, **B** GvHD-free, relapse-free survival (GRFS). GvHD relapse-free survival was calculated as the time from first HCT until the first occurrence of any of the following events: grades 3–4 aGvHD or moderate/severe cGvHD GF, disease relapse (poor graft function/graft failure with DADA2 disease relapse requiring repeat transplant), or death. Overall survival is calculated on total number of patients (*n* = 30); GRFS is calculated on total number of HCT procedures, excluding the two procedures performed with stem cells from an affected sibling (*n* = 34). **C** Effect of HCT on clinical features resolution. Black squares indicate death post-HCT. Dark gray squares represent the presence of a clinical feature/phenotype. Light gray squares represent major improvement in clinical features. White squares represent complete resolution of clinical features. Each patient is presented by 2 attached columns (before and after HCT) for comparison. Follow-up time post-HCT for each patient is shown in months (second row). Severe infections represent any viral, bacterial, or fungal infection that required antiviral or antifungal treatment or led to sepsis. FTT, failure to thrive; HSM, hepatosplenomegaly; LAP, lymphadenopathy

**Fig. 2:**
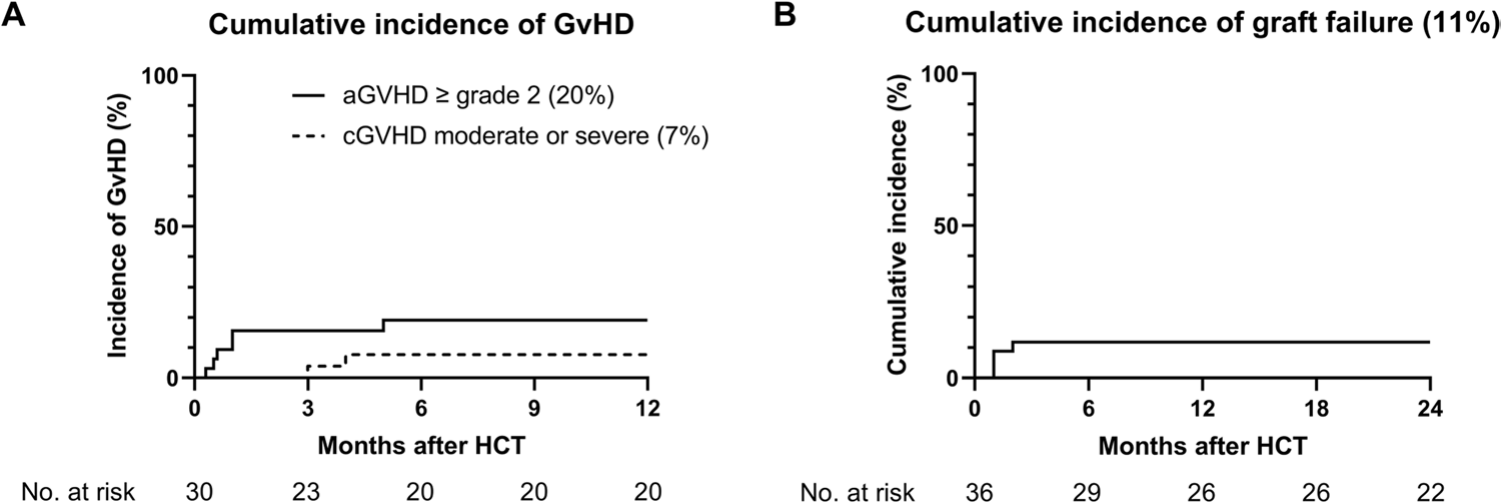
Kaplan–Meier curves representing **A** cumulative incidence of GvHD (a, acute grade 2 or higher; c, chronic moderate or severe); **B** cumulative incidence of graft failure. Cumulative incidence of GvHD and cumulative incidence of graft failure are calculated on total number of HCT procedures, excluding the two procedures performed with stem cells from an affected sibling (*n* = 34)

Median engraftment was d + 20 for neutrophils and d + 23 for platelets. In 25/28 patients receiving grafts from unaffected donors, full donor chimerism was achieved by d + 30. Overall survival at 2 years was 97%, with a median follow-up of survivors of 2 years (range: 0.5–16), accounting for 1545 patient-months post-HCT (Fig. 1A). P24 passed away 2 months post-HCT due to respiratory failure secondary to parainfluenza pneumonia despite full donor chimerism and treatment with steroids and etanercept for the suspicion of immune reconstitution inflammatory syndrome. Viral reactivation occurred in 17/30 patients (56%). Adenovirus, CMV, and BK were most frequent involved, each observed in six patients. GRFS was 73% at 2 years, with all events occurring within the first year post-HCT. Of those transplanted with affected donors (*n* = 2), P16 had primary graft failure requiring salvage HCT, and P29 had obtained near-full donor chimerism but failed erythroid line engraftment and remained with PRCA. Three patients experienced graft failure. P18 required two and P19 one subsequent HCT for secondary GF. The latter two patients were found to have aggregates of CD8 + T cells in their BM with low donor T cell chimerism (9% and 0%) (Fig. 1B, Fig. 2B). P28 required a second HCT likely due to low stem cell dose (1.4 × 10^6^/kg). P12 originally received MAC, but subsequently required two unconditioned HCTs due to drop in whole blood donor chimerism to 30% and new-onset RCA and agranulocytosis unresponsive to G-CSF. Cumulative incidence of aGvHD grades 2–4 was 20% at 1 year. Moderate-severe chronic GvHD developed in 2/30 patients (7%) at 1 year (Fig. 2A). P1, P19, and P24 developed sinusoidal obstruction syndrome (SOS), which responded to fluid restriction and diuresis in P1 and P19 and to defibrotide in P24. All three patients with SOS received either high-dose cyclophosphamide or myeloablative busulfan.

### Cure of DADA2

HCT cured the hematological phenotype in all patients, as confirmed at the most recent follow-up visit **(**Fig. 1C). No central vascular events were reported after engraftment (Fig. 1C). ADA2 plasma enzyme activity normalized in 16/17 patients tested post-HCT (Table 2). This normalization occurred as early as d + 12, coinciding with the reappearance of monocytes in the PB, as demonstrated by the prospective monitoring of plasma ADA2 enzyme activity in one patient [29]. At last follow-up, 29 patients were still alive, and 28 of these patients displayed full donor chimerism. Donor chimerism fell to 55% in P25 but remained stable over several months, with no evidence of disease. Transient hematological autoimmunity post-HCT was reported in three patients: ITP in two and AIHA in one. This autoimmunity responded to various treatment regimens (intravenous immunoglobulins (IVIG)/steroids/sirolimus/rituximab/bortezomib/romiplostim). Five patients are still on IVIG (one less than a year after HCT, one for mild bronchiectasis, and three post-rituximab treatment).

## Discussion

We show here that HCT for DADA2 cures the hematological and immunological phenotypes of DADA2, with no new vascular events, with excellent survival, after a median follow-up of 2 years. Outside this study, two additional patients with DADA2 have received HCT and are reported to be alive and well [30, 31]. Another two patients have also undergone HCT, one of whom died after receiving a graft from a donor heterozygous for the pathogenic mutation in *ADA2* (P. Stepensky, personal communication). Adding these patients to the current report, 32 of the 34 DADA2 patients who have undergone HCT were cured. Despite the temporary resolution of neutropenia by a multidrug approach (cyclosporine, steroids, adalimumab) in one patient prior to HCT, this option is not feasible in the long term, and HCT is, therefore, a valuable alternative. HCT also resolved the vascular phenotype in all 15 patients with vasculitis. No additional central nervous system vascular events were reported post-engraftment. Overall, the available data show an absence of new vascular events after engraftment post-HCT, demonstrating that hematopoietic cell-derived ADA2 plays a non-redundant role in restoring monocyte-endothelium interactions [12].

In the presence of this vascular phenotype, despite the impossibility of formal comparisons with other transplant indications, it is advisable to avoid high dose or untargeted busulfan and/or cyclophosphamide or high-dose radiation and to consider preventive measures for SOS. Liver disease was prominent and had a multifactorial etiology, directly related to DADA2 in some cases, but not in others (vasculitis, SOS, inflammation (DADA2/GvHD), iron overload, drug toxicity, GvHD). We therefore advise involving hepatologists in the care of DADA2 patients’ right from diagnosis, with imaging, functional assessment, liver biopsy, and iron chelation if indicated, and opting for the least hepatotoxic conditioning regimen. The evaluation of pre/post-HCT renal disease, although less well described, requires a similarly high level of attention.

Three patients who were grafted with a non-diseased donor, suffered GF. Low CD34^+^ stem cell dose probably contributed to GF of P28. The choice of conditioning may have contributed to secondary GF in P18 and P19, both of whom had low donor T cell chimerism preceding GF. Our data thus suggest that robust host lymphodepletion, more than myeloablation, is essential in preventing GF. It remains a matter of debate whether a related donor with a single deleterious allele of *ADA2* can be considered a suitable donor. Two of the six related donors carried heterozygous *ADA2* mutations (P3, P22: both HCT were successful), three were healthy (P1, P2, P21: all HCTs were successful), and one donor was of unknown status (P4). P25 has suffered no disease relapse, with a whole blood donor chimerism of 55%. In P12, whole blood donor chimerism fell to 30%, resulting in disease relapse, suggesting that there is a minimum required level of donor chimerism in DADA2. Indeed, patients carrying only a single pathogenic allele, with intermediate levels of ADA2 enzyme activity, have been reported to manifest DADA2 symptoms [10, 32]. In contrast, 9/10 MMUD and MUD may be considered suitable options for donors: There were 16 MUD and 8 MMUD transplants in this cohort.

The indication for HCT in this cohort was cytopenia and/or malignancy and immunodeficiency, not responding to treatment with TNF inhibitors. DADA2 patients with refractory BMF or immune cytopenia should be referred early on for HCT evaluation, given the morbidity and mortality due to hemorrhage, iron overload, infection, and long-term treatment with multiple immunosuppressive agents [18]. HCT is also a treatment option for patients who do not have access to anti-TNF agents even in the absence of immune cytopenia, BMF, or immunodeficiency. Theoretically, HCT could be a treatment option for patients on long-term treatment with anti-TNF inhibitors who develop neutralizing anti-drug antibodies [33]. Gene therapy (GT) is a promising option for the future, but is unlikely to be available to all DADA2 patients worldwide. In addition, GT requires conditioning and the presence of a sufficient number of autologous hematopoietic stem cells and may fail to reverse immune cytopenias if residual host T cells remain. Thus, GT is only feasible in patients without refractory cytopenia/BMF/malignancy, without prohibitive organ dysfunction, and without host T cell-mediated cytopenias. In the latter, HCT is likely the only potential curative treatment. For both HCT and GT, it emerges that full replacement of the host T cell compartment, and at least partial replacement of the myeloid compartment with ADA2 sufficient cells, is required.

In conclusion, we report here experience with the treatment of 30 DADA2 patients with cytopenia, BMF and immunodeficiency by HCT. All but one of the patients are alive and well and are cured at a median follow-up of 2 years. This successful treatment of an auto-inflammatory condition paves the way for application of HCT in other auto-inflammatory conditions refractory to classic immunosuppressive approaches.

## Supplementary Information

Below is the link to the electronic supplementary material.

Supplementary file1 (DOCX 19 KB)

## Data Availability

Available upon request to the corresponding authors.

## Abbreviations

HCT: Hematopoietic cell transplantation
DADA2: Deficiency of adenosine deaminase type 2
TNF-α: Tumor necrosis factor alpha
BMF: Bone marrow failure
PAN: Polyarteritis nodosa
GvHD: Graft-versus-host disease
GF: Graft failure
GRFS: GvHD-free relapse-free survival
MAC: Myeloablative conditioning
RIC: Reduced intensity conditioning
NMA: Non-myeloablative conditioning
PRCA: Pure red cell aplasia
MSD: HLA-matched sibling donor
MUD: HLA-matched unrelated donor
ATG: Anti-thymocyte globulin
