# Correction to: Hematopoietic Cell Transplantation Cures Adenosine Deaminase 2 Deficiency: Report on 30 Patients

**DOI:** 10.1007/s10875-022-01280-y

**Published:** 2022-04-30

**Authors:** Hasan Hashem, Giorgia Bucciol, Seza Ozen, Sule Unal, Ikbal Ok Bozkaya, Nurten Akarsu, Mervi Taskinen, Minna Koskenvuo, Janna Saarela, Dimana Dimitrova, Dennis D. Hickstein, Amy P. Hsu, Steven M. Holland, Robert Krance, Ghadir Sasa, Ashish R. Kumar, Ingo Müller, Monica Abreu de Sousa, Selket Delafontaine, Leen Moens, Florian Babor, Federica Barzaghi, Maria Pia Cicalese, Robbert Bredius, Joris van Montfrans, Valentina Baretta, Simone Cesaro, Polina Stepensky, Neven Benedicte, Despina Moshous, Guillaume Le Guenno, David Boutboul, Jignesh Dalal, Joel P. Brooks, Elif Dokmeci, Jasmeen Dara, Carrie L. Lucas, Sophie Hambleton, Keith Wilson, Stephen Jolles, Yener Koc, Tayfun Güngör, Caroline Schnider, Fabio Candotti, Sandra Steinmann, Ansgar Schulz, Chip Chambers, Michael Hershfield, Amanda Ombrello, Jennifer A. Kanakry, Isabelle Meyts

**Affiliations:** 1 Department of Pediatrics, Division of Pediatric Hematology and Oncology, Bone Marrow Transplant Unit, King Hussein Cancer Center (KHCC), P.O Box 1269, Amman, 11941 Jordan; 2 Department of Pediatrics, ERN RITA Core Center, University Hospitals Leuven, Herestraat 49, 3000 Louvain, Belgium; 3 Department of Microbiology, Immunology and Transplantation, Laboratory for Inborn Errors of Immunity, University Hospitals Leuven, Herestraat 49, 3000 Louvain, Belgium; 4 Department of Pediatric Rheumatology, Hacettepe University, Ankara, Turkey; 5 Hacettepe University Vasculitis Research Center, Ankara, Turkey; 6 Department of Pediatric Hematology, Research Center for Fanconi Anemia and Other Inherited Bone Marrow Failure Syndromes, Hacettepe University, Ankara, Turkey; 7 Division of Pediatric Hematology and Oncology, Bone Marrow Transplant Unit, University of Health Sciences, Ankara City Hospital, Ankara, Turkey; 8 Department of Medical Genetics, Hacettepe University, Sihhiye, 06100 Ankara, Turkey; 9 Division of Pediatric Hematology, Oncology and Stem Cell Transplantation, Helsinki University Hospital, Helsinki, Finland; 10 Pediatric Hematology, Oncology and Stem Cell Transplantation, Children and Adolescents, Helsinki University Hospital, Helsinki, Finland; 11 Institute for Molecular Medicine Finland, HiLIFE, University of Helsinki, Helsinki, Finland; 12 Centre for Molecular Medicine Norway, University of Oslo, Oslo, Norway; 13 Experimental Transplantation and Immunotherapy Branch, National Cancer Institute of the National Institutes of Health, Bethesda, MD USA; 14 Immune Deficiency Cellular Therapy Program, CCR, NCI, Bethesda, MD USA; 15 Laboratory of Clinical Infectious Diseases, National Institute of Allergy and Infectious Diseases, Bethesda, MD USA; 16 Cell and Gene Therapy, Baylor College of Medicine, Houston, TX USA; 17 Cincinnati Children’s Hospital Medical Center, Cincinnati, OH USA; 18 University of Cincinnati College of Medicine, Cincinnati, OH USA; 19 Division of Pediatric Stem Cell Transplantation and Immunology, University Medical Center Hamburg-Eppendorf, Hamburg, Germany; 20 Department of Pediatric Oncology, Hematology and Clinical Immunology, Center for Child and Adolescent Health, Medical Faculty, Heinrich-Heine-University, Dusseldorf, Germany; 21 San Raffaele Telethon Institute for Gene Therapy (TIGET), Pediatric Immunohematology and Bone Marrow Transplantation Unit, IRCCS San Raffaele Scientific Institute Milan, Milan, Italy; 22 Pediatric Immunohematology and Bone Marrow Transplantation Unit, IRCCS San Raffaele Scientific Institute, Milan, Italy; 23 Department of Pediatrics, Willem-Alexander Children’s Hospital, Leiden University Medical Center, Leiden, Netherlands; 24 Department of Pediatric Immunology and Infectious Diseases, Wilhelmina Children’s Hospital, University Medical Centre Utrecht, Utrecht, Netherlands; 25 Pediatric Hematology Oncology, Department of Mother and Child, Azienda Ospedaliera Universitaria Integrata, Verona, Italy; 26 Department of Bone Marrow Transplantation and Cancer Immunotherapy, Hadassah University Medical Center, Jerusalem, Israel; 27 Pediatric Immunology, Hematology and Rheumatology Unit, Hopital Necker-Enfants Malades, APHP, Paris, France; 28 Department of Internal Medicine, University Hospital Estaing, CHU Clermont-Ferrand, Clermont‑Ferrand, France; 29 Clinical Immunology Department, Hospital Saint Louis, Universite de Paris, Paris, France; 30 Rainbow Babies and Children’s Hospital, Case Western Reserve University, Cleveland, OH USA; 31 Department of Immunobiology, Yale University School of Medicine, New Haven, CT USA; 32 Department of Pediatrics, University of New Mexico, Albuquerque, NM USA; 33 Department of Pediatrics, Division of Allergy, Immunology, Blood and Marrow Transplantation, University of California San Francisco, San Francisco, CA USA; 34 Newcastle University Translational and Clinical Research Institute and Great North Children’s Hospital, Newcastle Upon Tyne Hospitals NHS Foundation Trust, Newcastle Upon Tyne, UK; 35 Department of Hematology, University Hospital of Wales, Cardiff, UK; 36 Immunodeficiency Centre for Wales, University Hospital of Wales, Cardiff, UK; 37 Stem Cell Transplant Unit, Medicana International, Istanbul, Turkey; 38 Division of Hematology/Oncology/Immunology, Gene Therapy, and Stem Cell Transplantation, University Children’s Hospital Zurich – Eleonore Foundation & Children’s Research Center (CRC), Steinwiesstrasse 75, CH‑8032 Zurich, Switzerland; 39 Pediatric Immuno‑Rheumatology of Western Switzerland, Department Women‑Mother‑Child, Lausanne University Hospital, Lausanne, Switzerland; 40 Division of Immunology and Allergy, Lausanne University Hospital and University of Lausanne, Lausanne, Switzerland; 41 Department of Pediatrics, University Medical Center Ulm, Ulm, Germany; 42 Vanderbilt University Medical Center, Nashville, TN USA; 43 Department of Medicine and Biochemistry, Duke University Medical Center, Durham, NC USA; 44 Metabolic, Cardiovascular, and Inflammatory Disease Genomics Branch, National Human Genome Research Institute (NHGRI), Bethesda, MD USA

**Correction to: Journal of Clinical Immunology (2021) 41:1633–1647**

10.1007/s10875-021-01098-0

This erratum is to clarify that the allele 1 of *ADA2* for patient 26 (P26) is p.Leu188Pro, and not p.Lys188Pro, as reported in Table 2.

