# Supplementary material for: Hematopoietic Cell Transplantation Cures Adenosine Deaminase 2 Deficiency: Report on 30 Patients
Source: J Clin Immunol. 2021 Jul 29;41(7):1633–47. doi: 10.1007/s10875-021-01098-0 (PMC8452581; doi:10.1007/s10875-021-01098-0)
Supplement: Supplementary file 1 — Supplementary file1 (DOCX 19 KB) [file 10875_2021_1098_MOESM1_ESM.docx]

**Online Supplement Journal of Clinical Immunology**

**Table S1:** Summary of patients and HCT characteristics

|  | **All patients /**  **All HCT procedures** | **Deceased** | **Poor graft function/**  **graft failure** |
| --- | --- | --- | --- |
| Number of patients | 30 | 1/30 (3%) | 6/30 (20%) |
| Number of HCT/procedures | 38 | 1/38 (2.5%) | 8/38 (21%)** |
| Median age at diagnosis, years (range) | 2.25 (0-16) | NA | 13 (2-18) |
| Median age at HCT, years (range) | 9 (2-28) | 9 | 5 (2-19) |
| M:F | 0.77:1 | F | 2.5:1 |
| Donor type |  |  |  |
| Healthy MSD | 1/38 (2.5%) | 0 | 0 |
| Healthy MRD | 3/38 (8%) | 0 | 0 |
| MSD from affected sibling | 2/38 (5.25%) | 0 | 2/2 (100%)** |
| MUD | 21/38 (55%) | 0 | 5/21 (24%) |
| MMUD | 9/38 (24%) | 1/9 (11%) | 1/9 (11%) |
| Haploidentical | 2/38 (5.25%) | 0 | 0 |
| Stem cell source |  |  |  |
| BM | 23/38 (60%) | 1/23 (4%) | 4/23 (17%)** |
| PB | 15/38 (40%) | 0 | 4/15 (26%) |
| Median CD34+ cell dose *10^6^/kg (n=26) (range) | 6 (0.5-15.45) | 5.6 | 5.2 (1.4-8.1) |
| Conditioning regimen |  |  |  |
| Cy - Bu | 5/38 (11%) | 0 | 2/5 (40%)** |
| Cy - Bu/Treo - Flu - TBI | 1/38 (5%) | 0 | 0 |
| Cy - Bu - Pentostatin | 2/38 (5%) | 0 | 2/2 (100%) |
| Cy - Flu | 1/38(2.5%) | 0 | 1/1 (100%) |
| Flu - Treo | 4/38(11%) | 0 | 1/4 (25%) |
| Flu - Treo - TT | 8/38 (21%) | 0 | 1/8 (12%) |
| Flu - Bu | 4/38 (11%) | 1/4 (25%) | 1/4 (25%) |
| Flu - Bu - TT | 2/38 (8%) | 0 | 0 |
| Flu - Mel | 2/38 (5%) | 0 | 0 |
| Flu - Mel - TT | 2/38 (5%) | 0 | 0 |
| Flu - TT | 1/38 (2.5%) | 0 | 0 |
| Flu - TBI | 2/38 (5%) | 0 | 0 |
| Flu | 2/38 (5.25%) | 0 | 0 |
| Serotherapy |  |  |  |
| Alemtuzumab | 17/38 (45%) | 1/17 (6%) | 3/17 (18%)** |
| ATG | 12/38 (32%) | 0 | 3/12 (25%)** |
| None | 9/38 (23%) | 0 | 2/9 (22%) |
| GvHD prophylaxis |  |  |  |
| CSA | 3/38 (8%) | 0 | 1/3 (33%)** |
| CSA + MTX | 13/38 (34%) | 0 | 1/13 (8%) |
| CSA + MMF | 8/38 (29%) | 1/8 (12.5%) | 2/8 (25%) |
| CSA + prednisone | 2/38 (5.5%) | 0 | 0 |
| MMF | 1/38 (2.5%) | 0 | 0 |
| Tacrolimus + MTX | 2/38 (5.5%) | 0 | 1/2 (50%)** |
| Tacrolimus + MMF | 4/38 (10.5%) | 0 | 1/4 (25%) |
| Sirolimus + MMF | 1/38 (2.5%) | 0 | 1/1 (100%) |
| Additional PTCy | 6/38 (16%) | 1/6 (16.5%) | 2/6 (33%) |

**including patients transplanted with BM from an affected sibling. Two procedures were performed without conditioning (stem cell boosts). ATG: anti-thymocyte globulins; BM: bone marrow; Bu: busulfan; CSA: cyclosporine A; Cy: cyclophosphamide; F: female; Flu: fludarabine; GvHD: graft versus host disease; HCT: hematopoietic stem cell transplantation; M: male; Mel: melphalan; MMF: mycophenolate mofetil; MMUD: mismatched unrelated donor; MSD: matched sibling donor; MTX: methotrexate; MUD: matched unrelated donor; PB: peripheral blood; Pento: pentostatin; PTCy: post-transplant cyclophosphamide; TBI: total body irradiation; Treo: treosulfan; TT: thiotepa.
